# Clean and Efficient Synthesis of Isoxazole Derivatives in Aqueous Media

**DOI:** 10.3390/molecules181113645

**Published:** 2013-11-05

**Authors:** Guolan Dou, Pan Xu, Qiang Li, Yukun Xi, Zhibin Huang, Daqing Shi

**Affiliations:** 1 School of Safety Engineering, China University of Mining & Technology, Xuzhou 221116, China; 2 Key Laboratory of Organic Synthesis of Jiangsu Province, College of Chemistry, Chemical Engineering and Materials Science, Soochow University, Suzhou 215123, China; 3 Hainan Chuntch Pharmaceutical Company Limited, Hainan Province Seaport Bonded Area 6th Workshop, Haikou 570216, China

**Keywords:** 5-arylisoxazole, without catalyst, aqueous media, synthesis

## Abstract

A series of 5-arylisoxazole derivatives were synthesized via the reaction of 3-(dimethyl-amino)-1-arylprop-2-en-1-ones with hydroxylamine hydrochloride in aqueous media without using any catalyst. This method has the advantages of easier work-up, mild reaction conditions, high yields, and an environmentally benign procedure.

## 1. Introduction

The need to reduce the amount of toxic waste and byproducts arising from chemical processes requires increasing emphasis on the use of less toxic and environmentally compatible materials in the design of new synthetic methods [1]. One of the most promising approaches is the use of water as the reaction medium [2]. Compared to organic solvents the aqueous medium is less expensive, less dangerous, and more environmentally friendly. In recent years, there has been increasing recognition that water is an attractive medium for many organic reactions [3,4,5]. Many important types of heterocycles, such as furans, pyridines, quinolines, indoles, triazines, acridines, pyrazines, and pyrimidines have been synthesized in aqueous media [6,7,8,9,10,11,12,13,14,15]. The synthesis of new and other important type of heterocyclic compounds in water continues to attract wide attention among synthetic chemists.

Nitrogen-containing heterocyclic building blocks are of great importance to both medical and organic chemists, and their synthesis continues to represent a challenge from both academic and industrial perspectives [16]. Isoxazole derivatives are an important class of heterocyclic pharmaceuticals and bioactive natural products because of their significant and wide spectrum of biological activities, including potent and selective antagonism of the NMDA receptor [17] and anti-HIV activity [18]. Many syntheses of isoxazoles have been developed [19,20]. However, these syntheses are usually carried out in organic solvents. As part of our current studies on the development of new routes to heterocyclic systems in aqueous media [21,22,23,24,25,26,27,28], we now report an efficient and clean synthetic route to isoxazole derivatives via the reaction of 3-(dimethylamino)-1-arylprop-2-en-1-ones with hydroxylamine hydrochloride in aqueous media.

## 2. Results and Discussion

**Scheme 1:**
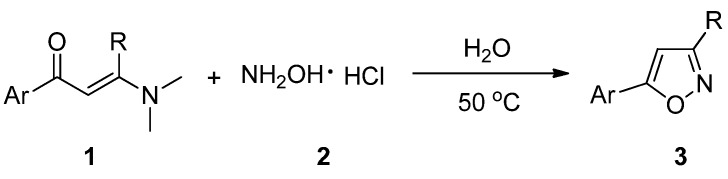
The synthesis of 5-arylisoxazole derivatives in aqueous media.

When an equivalent mixture of an 3-(dimethylamino)-1-arylprop-2-en-1-one derivative **1** and hydroxylamine hydrochloride (**2**) was stirred at 50 °C in aqueous media, 5-arylisoxazole derivatives **3** were obtained in good yields (Scheme 1). The results are summarized in Table 1.

**Table 1:** The synthetic results of 5-arylisoxazole derivatives in aqueous media.

Entry	Product	Ar	R	Isolated Yield (%)
1	**3a**	4-ClC_6_H_4_	H	88
2	**3b**	4-CH_3_OC_6_H_4_	H	93
3	**3c**	4-BrC_6_H_4_	H	89
4	**3d**	Naphthen-2-yl	H	84
5	**3e**	C_6_H_5_	CH_3_	88
6	**3f**	4-CH_3_OC_6_H_4_	CH_3_	92
7	**3g**	4-CH_3_C_6_H_4_	CH_3_	89
8	**3h**	4-BrC_6_H_4_	CH_3_	90
9	**3i**	4-ClC_6_H_4_	CH_3_	86
10	**3j**	4-CH_3_OCOC_6_H_4_	CH_3_	84
11	**3k**	4-BocNHC_6_H_4_	CH_3_	86
12	**3l**	Thiophen-2-yl	CH_3_	93

As shown in Table 1, this protocol could be applied to the 3-(dimethylamino)-1-arylprop-2-en-1-ones with both electron-withdrawing groups (such as halide groups) and electron-donating groups (such as methyl or methoxyl groups). Polysubstituted 3-(dimethylamino)-1-arylprop-2-en-1-ones could also be used in this synthesis. We concluded that the electronic nature of the substituent on the aromatic ring of 3-(dimethylamino)-1-arylprop-2-en-1-ones had no significant effect on this reaction. This synthesis was confirmed to follow the group-assisted-purification chemistry process [29,30,31], which can avoid traditional chromatography and recrystallization purification, that is, all the pure products can be obtained only by suction filtration without further purification. All the products **3** were identified from their IR, ^1^H-NMR, and HRMS spectra.

**Scheme 2:**
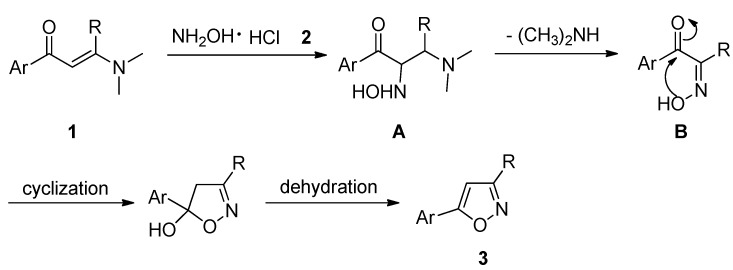
The proposed mechanism for the synthesis of 5-arylisoxazoles.

Although the detailed mechanism of this reaction remains to be fully clarified, the formation of 5-arylisoxazoles **3** could be explained by the reaction sequence presented in Scheme 2. First, the Michael addition of 3-(dimethylamino)-1-arylprop-2-en-1-ones **1** and hydroxylamine **2** gives the intermediate **A**, which then eliminates one molecule of dimethylamine to give the intermediate **B**, which upon intramolecular cyclization and dehydration gives rise to the final product **3**.

## 3. Experimental

All reagents were purchased from commercial suppliers and used without further purification. Melting points are uncorrected. IR spectra were recorded on Varian F-1000 spectrometer in KBr with absorptions in cm^−1^. ^1^H-NMR and ^13^C-NMR spectra were recorded on a Varian Inova-300 MHz or Varian Inova-400 MHz in CDCl_3_ solution. *J* Values are in Hertz. Chemical shifts are expressed in parts per million downfield from internal standard TMS. High-resolution mass spectra (HRMS) were obtained using Bruker microTOF-Q instrument.

### 3.1. General Procedure for the Synthesis of 3-(Dimethylamino)-1-arylprop-2-en-1-ones **1a**–**l**

A solution of substituted acetophenone (2 nmol) in *N*,*N*-dimethylformamide dimethyl acetal or *N*,*N*-dimethylacetamide dimethyl acetal (10 mL) was refluxed for 20 h during which time some methanol was formed and removed through a reflux condenser. After cooling, the precipitate was collected by suction to give compounds **1**.

*1-(4-Chlorophenyl)-3-(dimethylamino)prop-2-en-1-one* (**1a**). Mp: 84–86 °C; IR (KBr) *ν*: 2916, 2804, 1648, 1580, 1545, 1433, 1411, 1353, 1279, 1237, 1118, 1088, 1053, 1010, 980, 899, 837, 790, 742, 678 cm^−1^; ^1^H-NMR (400 MHz, CDCl_3_) δ (ppm) 2.89 (s, 3H, NCH_3_), 3.12 (s, 3H, NCH_3_), 5.63 (d, *J* = 12.4 Hz, 1H, CH), 7.34 (d, *J* = 8.4 Hz, 2H, ArH), 7.78 (d, *J* = 12.8 Hz, 1H, CH), 7.81 (d, *J* = 8.4 Hz, 2H, ArH); ^13^C-NMR (75 MHz, CDCl_3_) δ (ppm) 37.2, 45.0, 91.6, 128.2, 128.8, 136.8, 138.7, 154.4, 187.0; HRMS calcd. for C_11_H_13_ClNO [M+H]^+^: 210.0686; found: 210.0685.

*3-(Dimethylamino)-1-(4-methoxyphenyl)prop-2-en-1-one* (**1b**). Mp: 92–94 °C; IR (KBr) *ν*: 2905, 2838, 1643, 1603, 1583, 1432, 1358, 1305, 1242, 1175, 1117, 1057, 1026, 901, 774 cm^−1^; ^1^H-NMR (400 MHz, CDCl_3_) δ (ppm) 2.89 (s, 3H, NCH_3_), 3.05 (s, 3H, NCH_3_), 3.80 (s, 3H, CH_3_O), 5.66 (d, *J* = 12.4 Hz, 1H, CH), 6.86-6.88 (m, 2H, ArH), 7.74 (d, *J* = 12.4 Hz, 1H, CH), 7.86–7.88 (m, 2H, ArH); ^13^C-NMR (75 MHz, CDCl_3_) δ (ppm) 37.1, 44.8, 55.2, 91.5, 113.1, 129.3, 132.9, 153.7, 161.8, 187.2; HRMS calcd. for C_12_H_16_NO_2_ [M+H]^+^: 206.1181; found: 206.1205.

*1-(4-Bromophenyl)-3-(dimethylamino)prop-2-en-1-one* (**1c**). Mp: 81–83 °C; IR (KBr) *ν*: 2910, 2808, 1649, 1575, 1540, 1435, 1357, 1304, 1271, 1237, 1120, 1056, 1003, 895, 847, 808, 775, 760, 673 cm^−1^; ^1^H-NMR (400 MHz, CDCl_3_) δ (ppm) 2.88 (s, 3H, NCH_3_), 3.11 (s, 3H, NCH_3_), 5.62 (d, *J* = 12.4 Hz, 1H, CH), 7.50 (d, *J* = 8.4 Hz, 2H, ArH), 7.73 (s, 1H, CH), 7.75–7.79 (m, 2H, ArH); ^13^C-NMR (75 MHz, CDCl_3_) δ (ppm) 37.3, 45.1, 91.6, 125.4, 129.1, 131.3, 139.3, 154.6, 187.2; HRMS calcd. for C_11_H_13_BrNO [M+H]^+^: 254.0181; found: 254.0182.

*3-(Dimethylamino)-1-(naphthalen-2-yl)prop-2-en-1-one* (**1d**). Mp: 94–95 °C; IR (KBr) *ν*: 2927, 2898, 2809, 1636, 1554, 1427, 1291, 1253, 1189, 1111, 1045, 909, 863, 826, 781, 759 cm^−1^; ^1^H-NMR (400 MHz, CDCl_3_) δ (ppm) 2.91 (s, 3H, NCH_3_), 3.09 (s, 3H, NCH_3_), 5.85 (d, *J* = 12.4 Hz, 1H, CH), 7.46–7.53 (m, 2H, ArH), 7.82–7.87 (m, 3H, ArH), 7.92 (t, *J* = 6.8 Hz, 1H, CH), 8.00–8.03 (m, 1H, ArH), 8.40 (s, 1H, ArH); ^13^C-NMR (75 MHz, CDCl_3_) δ (ppm) 37.3, 45.0, 92.4, 124.7, 126.2, 127.2, 127.7, 127.8, 129.2, 132.8, 134.7, 137.9, 154.3, 188.4; HRMS calcd. for C_15_H_15_NO [M+H]^+^: 226.1232; found: 226.1234.

*3-(Dimethylamino)-1-phenylbut-2-en-1-one* (**1e**). Oil; IR (KBr) *ν*: 2911, 1650, 1555, 1500, 1450, 1357, 1310, 1267, 1027, 1067, 1000, 900, 857, 775, 761, 673 cm^−1^; ^1^H-NMR (400 MHz, CDCl_3_) δ (ppm) 2.64 (s, 3H, CH_3_), 3.03 (s, 6H, N(CH_3_)_2_), 5.69 (s, 1H, CH), 7.13 (s, 1H, ArH), 7.41 (t, *J* = 6.4 Hz, 2H, ArH), 7.82 (d, *J* = 7.6 Hz, 2H, ArH); ^13^C-NMR (75 MHz, CDCl_3_) δ (ppm) 19.5, 42.7, 52.4, 117.3, 128.2, 130.0, 130.5, 133.9, 151.6, 166.1, 172.3; HRMS calcd. for C_12_H_15_NO [M+H]^+^: 190.1232; found: 190.1245.

*3-(Dimethylamino)-1-(4-methoxyphenyl)but-2-en-1-one* (**1f**). Mp: 134–136 °C; IR (KBr) *ν*: 2961, 2835, 1671, 1574, 1460, 1307, 1167, 1081, 1025, 917, 865, 782, 746, 694 cm^−1^; ^1^H-NMR (400 MHz, CDCl_3_) δ (ppm) 2.62 (s, 3H, CH_3_), 3.02 (s, 6H, N(CH_3_)_2_), 3.81 (s, 3H, CH_3_O), 5.64 (s, 1H, CH), 6.86 (d, *J* = 8.4 Hz, 2H, ArH), 7.84 (d, *J* = 8.4 Hz, 2H, ArH); ^13^C-NMR (75 MHz, CDCl_3_) δ (ppm) 16.3, 39.9, 55.2, 92.1, 113.0, 129.0, 135.5, 161.3, 163.3, 187.1; HRMS calcd. for C_13_H_18_NO_2_ [M+H]^+^: 220.1338; found: 220.1341.

*3-(Dimethylamino)-1-(-4-methylphenyl)but-2-en-1-one* (**1g**). Mp: 94–96 °C; IR (KBr) *ν*: 2913, 2808, 1660, 1577, 1561, 1450, 1357, 1300, 1277, 1122, 1066, 1000, 899, 847, 775, 760, 673 cm^−1^; ^1^H-NMR (400 MHz, CDCl_3_) δ (ppm) 2.37 (s, 3H, CH_3_), 2.65 (s, 3H, CH_3_), 3.06 (s, 6H, N(CH_3_)_2_), 5.67 (s, 1H, CH), 7.18 (d, *J* = 7.6 Hz, 2H, ArH), 7.77 (d, *J* = 8.0 Hz, 2H, ArH); ^13^C-NMR (75 MHz, CDCl_3_) δ (ppm) 16.4, 21.4, 40.0, 92.6, 127.3, 128.7, 140.3, 140.5, 163.6, 188.1; HRMS calcd. for C_13_H_17_NO [M+H]^+^: 204.1388; found: 204.1386.

*1-(4-Bromophenyl)-3-(dimethylamino)but-2-en-1-one* (**1h**). Mp: 88–92 °C; IR (KBr) *ν*: 2931, 1721, 1616, 1500, 1420, 1385, 1354, 1220, 1161, 1069, 1030, 1007, 849, 769, 680, 627 cm^−1^; ^1^H-NMR (400 MHz, CDCl_3_) δ (ppm) 2.63 (s, 3H, CH_3_), 3.06 (s, 6H, N(CH_3_)_2_), 5.58 (s, 1H, CH), 7.49 (d, *J* = 8.4 Hz, 2H, ArH), 7.70 (d, *J* = 8.4 Hz, 2H, ArH); ^13^C-NMR (75 MHz, CDCl_3_) δ (ppm) 16.5, 40.1, 92.0, 124.6, 128.4, 128.9, 131.1, 131.8, 141.8, 186.7; HRMS calcd. for C_12_H_14_BrNO [M+H]^+^: 267.0259; found: 267.0256.

*1-(4-Chlorophenyl)-3-(dimethylamino)but-2-en-1-one* (**1i**). Mp: 82–84 °C; IR (KBr) *ν*: 3039, 2961, 2804, 1676, 1620, 1537, 1411, 1379, 1351, 1278, 1224, 1166, 1089, 1024, 1010, 921, 862, 772, 739, 712, 678 cm^−1^; ^1^H-NMR (400 MHz, CDCl_3_) δ (ppm) 2.62 (s, 3H, CH_3_), 3.04 (s, 6H, N(CH_3_)_2_), 5.57 (s, 1H, CH), 7.31 (d, *J* = 8.4 Hz, 2H, ArH), 7.76 (d, *J* = 8.4 Hz, 2H, ArH); ^13^C-NMR (75 MHz, CDCl_3_) δ (ppm) 16.4, 40.1, 92.0, 128.0, 128.6, 136.0, 141.3, 164.3, 186.5; HRMS calcd. for C_12_H_14_ClNO [M+H]^+^: 224.0842; found: 224.0861.

*Methyl 4-(3-dimethylamino)but-2-enoyl)benzoate* (**1j**). Mp: 103–106 °C; IR (KBr) *ν*: 2951, 1720, 1620,1600, 1569, 1434, 1282, 1217, 1110, 1037, 1011, 921, 868, 825, 759, 725 cm^−1^; ^1^H-NMR (400 MHz, CDCl_3_) δ (ppm) 2.64 (s, 3H, CH_3_), 3.06 (s, 6H, N(CH_3_)_2_), 3.89 (s, 3H, CH_3_), 5.61 (s, 1H, CH), 7.85 (d, *J* = 8.0 Hz, 2H, ArH), 8.01 (d, *J* = 8 Hz, 2H, ArH); ^13^C-NMR (75 MHz, CDCl_3_) δ (ppm) 16.5, 40.1, 52.1, 92.5, 127.1, 129.3, 131.2, 147.0, 164.6, 166.7, 186.9; HRMS calcd. for C_14_H_17_NO_3_ [M+H]^+^: 248.1287; found: 248.1292.

*tert-Butyl (4-(3-dimethylamino)but-2-enoyl)phenyl)carbamate* (**1k**). Mp: 218–220 °C; IR (KBr) *ν*: 3242, 3087, 2963, 1721, 1615, 1482, 1361, 1310, 1269, 1245, 1152, 1085, 1050, 1022, 922, 866, 788, 690 cm^−1^; ^1^H-NMR (400 MHz, CDCl_3_) δ (ppm) 1.50 (s, 9H, C(CH_3_)_3_), 2.62 (s, 3H, CH_3_), 3.04 (s, 6H, N(CH_3_)_2_), 5.65 (s, 1H, CH), 6.83 (s, 1H, NH), 7.37 (d, *J* = 8.4 Hz, 2H, ArH), 7.81 (d, *J* = 8.8 Hz, 2H, ArH); ^13^C-NMR (75 MHz, CDCl_3_) δ (ppm) 16.4, 28.3, 40.0, 80.6, 92.3, 117.3, 128.4, 137.4, 140.4, 152.5, 163.5, 187.2; HRMS calcd. for C_17_H_25_N_2_O_3_ [M+H]^+^: 305.1865; found: 305.1866.

*3-(Dimethylamino)-1-(thiophen-2-yl)but-2-en-1-one* (**1l**). Mp: 90–91 °C; IR (KBr) *ν*: 3078, 2919, 2815, 1694, 1622, 1543, 1422, 1380, 1345, 1228, 1170, 1085, 1064, 1028, 906, 860, 837, 771, 723 cm^−1^; ^1^H-NMR (400 MHz, CDCl_3_) δ (ppm) 2.59 (s, 3H, CH_3_), 3.01 (s, 6H, N(CH_3_)_2_), 5.60 (s, 1H, CH), 6.99 (s, 1H, ArH), 7.37 (s, 1H, ArH), 7.51 (s, 1H, ArH); ^13^C-NMR (75 MHz, CDCl_3_) δ (ppm) 16.5, 40.0, 91.6, 127.2, 127.4, 129.4, 150.0, 163.8, 180.1; HRMS calcd. for C_10_H_14_NOS [M+H]^+^: 196.0796; found: 196.0807.

### 3.2. General Procedure for the Synthesis of Isoxazole Derivatives **3a**–**l**

3-(Dimethylamino)-1-arylprop-2-en-1-one **1** (1 nmol), hydroxylamine hydrochloride **2** (1 nmol) and water (5 mL) were added to a 25-mL round-bottom flask. The mixture was then stirred at 50 °C for 2 h. After completion of the reaction, the mixture was then cooled to room temperature. The precipitate was collected by suction filtration to give products **3** without further purification.

*5-(4-Chlorophenyl)isoxazole* (**3a**). Mp: 85–87 °C (lit. [19] 84–85 °C); IR (KBr) *ν*: 1601, 1447, 1264, 1128, 1109, 1088, 802 cm^−1^; ^1^H-NMR (400 MHz, CDCl_3_) δ (ppm) 6.52 (d, *J* = 2.0 Hz, 1H, C^4^-H), 7.45 (d, *J* = 8.4 Hz, 2H, ArH), 7.73 (d, *J* = 8.4 Hz, 2H, ArH), 8.30 (d, *J* = 2.0 Hz, 1H, C^3^-H); ^13^C-NMR (75 MHz, CDCl_3_) δ (ppm) 98.9, 125.6, 127.0, 129.2, 136.1, 150.8, 168.1; HRMS calcd. for C_9_H_7_ClNO [M+H]^+^: 180.0216; found: 180.0215.

*5-(4-Methoxyphenyl)isoxazole* (**3b**). Mp: 60–62 °C (lit. [19] 64–65 °C); IR (KBr) *ν*: 3002, 1605, 1510, 1446, 1251, 1175, 1020, 906, 787, 675 cm^−1^; ^1^H-NMR (400 MHz, CDCl_3_) δ (ppm) 3.86 (s, 3H, CH_3_O), 6.39 (d, *J* = 1.6 Hz, 1H, C^4^-H), 6.98 (d, *J* = 8.8 Hz, 2H, ArH), 7.73 (d, *J* = 8.8 Hz, 2H, ArH), 8.25 (d, *J* = 1.6 Hz, 1H, C^3^-H); ^13^C-NMR (75 MHz, CDCl_3_) δ (ppm) 55.3, 97.2, 114.3, 120.0, 127.3, 150.7, 161.0, 169.2; HRMS calcd. for C_10_H_10_NO_2_ [M+H]^+^: 176.0712; found: 176.0718.

*5-(4-Bromophenyl)isoxazole* (**3c**). Mp: 112–114 °C (lit. [19] 114–116 °C); IR (KBr) *ν*: 1629, 1427, 1077, 1021, 846, 775 cm^−1^; ^1^H-NMR (400 MHz, CDCl_3_) δ (ppm) 6.53 (d, *J* = 1.6 Hz, 1H, C^4^-H), 7.60 (d, *J* = 8.4 Hz, 2H, ArH), 7.66 (d, *J* = 8.8 Hz, 2H, ArH), 8.30 (d, *J* = 1.6 Hz, 1H, C^3^-H); ^13^C-NMR (75 MHz, CDCl_3_) δ (ppm) 99.0, 124.5, 126.0, 127.2, 132.2, 150.9, 168.2; HRMS calcd. for C_9_H_7_BrNO [M+H]^+^: 223.9711; found: 223.9716.

*5-(Naphthalen-1-yl)isoxazole* (**3d**). Mp: 90–92 °C; IR (KBr) *ν*: 3049, 1562, 1449, 1360, 1265, 1190, 910, 808, 738 cm^−1^; ^1^H-NMR (300 MHz, CDCl_3_) δ (ppm) 6.63 (d, *J* = 1.6 Hz, 1H, C^4^-H), 7.53–7.56 (m, 2H, ArH), 7.84–7.93 (m, 4H, ArH), 8.30–8.34 (m, 2H, C^3^-H and ArH); ^13^C-NMR (75 MHz, CDCl_3_) δ (ppm) 98.9, 122.8, 124.3, 125.5, 126.8, 127.2, 127.7, 128.5, 128.8, 132.9, 133.8, 150.8, 169.3; HRMS calcd. for C_13_H_10_NO [M+H]^+^: 196.0762; found: 196.0768.

*3-Methyl-5-phenylisoxazole* (**3e**). Mp: 67–69 °C (lit. [32] 67 °C); IR (KBr) *ν*: 3056, 2983, 1600, 1424, 1258, 1039, 897, 766, 683 cm^−1^; ^1^H-NMR (400 MHz, CDCl_3_) δ (ppm) 2.36 (s, 3H, CH_3_), 6.37 (s, 1H, C^4^-H), 7.42–7.48 (m, 3H, ArH), 7.75–7.77 (m, 2H, ArH); ^13^C-NMR (75 MHz, CDCl_3_) δ (ppm) 11.4, 100.1, 125.6, 127.4, 128.8, 129.9, 160.2, 169.5; HRMS calcd. for C_10_H_10_NO [M+H]^+^: 160.0762; found: 160.0770.

*5-(4-Methoxyphenyl)-3-methylisoxazole* (**3f**). Mp: 99–101 °C; IR (KBr) *ν*: 2936, 1612, 1510, 1430, 1253, 1174, 1022, 787, 680 cm^−1^; ^1^H-NMR (400 MHz, CDCl_3_) δ (ppm) 2.33 (s, 3H, CH_3_), 3.85 (s, 3H, CH_3_O), 6.24 (s, 1H, C^4^-H), 6.96 (d, *J* = 8.8 Hz, 2H, ArH), 7.69 (d, *J* = 8.8 Hz, 2H, ArH); ^13^C-NMR (75 MHz, CDCl_3_) δ (ppm) 11.4, 55.2, 98.7, 114.2, 120.3, 127.2, 160.2, 160.8, 169.5; HRMS calcd. for C_11_H_12_NO_2_ [M+H]^+^: 190.0868; found: 190.0862.

*3-Methyl-5-(4-methylphenyl)isoxazole* (**3g**). Mp: 88–90 °C (lit. [33] 92 °C); IR (KBr) *ν*: 3063, 2961, 1603, 1415, 1259, 1114, 1044, 956, 895, 792, 680 cm^−1^; ^1^H-NMR (400 MHz, CDCl_3_) δ (ppm) 2.13 (s, 3H, CH_3_), 2.18 (s, 3H, CH_3_), 6.09 (s, 1H, C^4^-H), 7.04 (d, *J* = 8.4 Hz, 2H, ArH), 7.43 (d, *J* = 8.0 Hz, 2H, ArH); ^13^C-NMR (75 MHz, CDCl_3_) δ (ppm) 11.4, 21.3, 99.5, 124.8, 125.6, 129.5, 140.1, 160.2, 169.7; HRMS calcd. for C_11_H_12_NO [M+H]^+^: 174.0919; found: 174.0914.

*5-(4-Bromophenyl)-3-methylisoxazole* (**3h**). Mp: 126–128 °C; IR (KBr) *ν*: 2978, 1598, 1467, 1404, 1256, 1061, C^4^-H), 7.41 (d, *J* = 8.0 Hz, 2H, ArH), 7.67 (d, *J* = 8.0 Hz, 2H, ArH); ^13^C-NMR (75 MHz, CDCl_3_) δ (ppm) 11.4, 100.4, 125.9, 126.9, 129.1, 135.9, 160.4, 168.4; HRMS calcd. for C_10_H_9_ClNO [M+H]^+^: 194.0373; found: 194.0378.

*5-(4-Chlorophenyl)-3-methylisoxazole* (**3i**). Mp: 88–89 °C (lit. [34] 90–91 °C); IR (KBr) *ν*: 1600, 1451, 1400, 1249, 1092, 1053, 833cm^-1^; ^1^H-NMR (400 MHz, CDCl_3_) δ (ppm) 2.34 (s, 3H, CH_3_), 6.34 (s, 1H, C^4^-H), 7.41 (d, *J* = 8.0 Hz, 2H, ArH), 7.67 (d, *J* = 8.0 Hz, 2H, ArH); ^13^C-NMR (75 MHz, CDCl_3_) δ (ppm) 11.4, 100.4, 125.9, 126.9, 129.1, 135.9, 160.4, 168.4; HRMS calcd. for C_10_H_9_ClNO [M+H]^+^: 194.0373; found: 194.0378.

*Methyl 4-(3-methylisoxazol-5-yl)benzoate* (**3j**). Mp: 88–90 °C; IR (KBr) *ν*: 2945, 1720, 1601, 1419, 1274, 1182, 1105, 950, 774 cm^−1^; ^1^H-NMR (400 MHz, CDCl_3_) δ (ppm) 2.36 (s, 3H, CH_3_), 3.93 (s, 3H, CH_3_O), 6.46 (s, 1H, C^4^-H), 7.81 (d, *J* = 8.4 Hz, 2H, ArH), 8.10 (d, *J* = 8.4 Hz, 2H, ArH); ^13^C-NMR (75 MHz, CDCl_3_) δ (ppm) 11.4, 52.2, 101.6, 125.5, 130.1, 131.1, 131.2, 160.4, 166.2, 168.3; HRMS calcd. for C_12_H_12_NO_3_ [M+Na]^+^: 240.0637; found: 240.0650.

*tert-Butyl 4-(3-methylisoxazol-5-yl)phenylcarbamate* (**3k**). Mp: 120–121 °C; IR (KBr) *ν*: 3363, 3005, 2978, 1701, 1520, 1413, 1237, 1160, 835, 772 cm^−1^; ^1^H-NMR (400 MHz, CDCl_3_) δ (ppm) 1.48 (s, 9H, (CH_3_)_3_C), 2.25 (s, 3H, CH_3_), 6.71 (s, 1H, C^4^-H), 7.59 (d, *J* = 8.8 Hz, 2H, ArH), 7.71 (d, *J* = 8.4 Hz, 2H, ArH), 9.65 (s, 1H, NH); ^13^C-NMR (75 MHz, CDCl_3_) δ (ppm) 11.5, 28.2, 80.9, 99.2, 118.3, 122.1, 126.6, 140.0, 152.4, 160.3, 169.3; HRMS calcd. for C_15_H_19_N_2_O_3_ [M+H]^+^: 275.1396; found: 275.1392.

*3-Methyl-5-(thiophen-2-yl)isoxazole* (**3l**). Oil; IR (KBr) *ν*: 2933, 1605, 1422, 1033, 899, 792, 706 cm^−1^; ^1^H-NMR (400 MHz, CDCl_3_) δ (ppm) 2.33 (s, 3H, CH_3_), 6.24 (s, 1H, C^4^-H), 7.11 (t, *J* = 4.4 Hz, 1H, ArH), 7.43 (d, *J* = 4.8 Hz, 1H, ArH), 7.48 (d, *J* = 3.6 Hz, 1H, ArH); ^13^C-NMR (75 MHz, CDCl_3_) δ (ppm) 11.4, 100.0, 126.7, 127.7, 128.0, 129.4, 160.3, 160.4; HRMS calcd. for C_8_H_8_NOS [M+H]^+^: 166.0327; found: 166.0322.

## 4. Conclusions

In conclusion, we have developed an efficient synthesis of isoxazole derivatives via the reaction of 3-(dimethylamino)-1-arylprop-2-en-1-ones with hydroxylamine hydrochloride in aqueous media without using any catalyst. This method has the advantages of an easier work-up, mild reaction conditions, high yields, and an environmentally benign procedure.
